# Change in central corneal thickness, corneal endothelial cell density, anterior chamber depth and axial length after repair of rhegmatogenous retinal detachment

**DOI:** 10.12669/pjms.336.13584

**Published:** 2017

**Authors:** Ahsan Mukhtar, Mohammad Asim Mehboob, Zaheer Uddin Babar, Mazhar Ishaq

**Affiliations:** 1 Dr. Ahsan Mukhtar, MCPS, FCPS(Ophth), FCPS (Vitreoretina), FRCS. Armed Forces Institute of Ophthalmology, Rawalpindi, Pakistan; 2 Dr. Mohammad Asim Mehboob, FCPS (Ophth), MRCSEd (Ophth) PNS Shifa Naval Hospital, Karachi, Pakistan. Armed Forces Institute of Ophthalmology, Rawalpindi, Pakistan; 3 Dr. Zaheer Uddin Babar, MCPS, FCPS(Ophth). PAF Hospital, Rafiqi Base, Shorkot, Pakistan. Armed Forces Institute of Ophthalmology, Rawalpindi, Pakistan; 4 Prof. Dr. Mazhar Ishaq, FRCOPHTH, FCPS(Ophth). Armed Forces Institute of Ophthalmology, Rawalpindi, Pakistan

**Keywords:** Anterior Chamber Depth, Axial Length, Corneal Endothelial cell density, Pars plana Vitrectomy, Rhegmatogenous Retinal Detachment, Scleral Buckling

## Abstract

**Objective:**

To compare the effects of Pars Plana Vitrectomy (PPV) and Scleral Buckling (SB) with reference to Central Corneal Thickness (CCT), Corneal Endothelial-Cell Density (CED), Anterior Chamber Depth (ACD) and Axial Length (AL) in repair of Rhegmatogenous Retinal Detachment (RRD).

**Methods:**

This comparative cross sectional analytical study was conducted at Armed Forces Institute of Ophthalmology (AFIO), Rawalpindi from July 2013 to July 2015. A total of 69 eyes of 69 patients which underwent repair of RRD by either PPV or SB were analyzed to compare mean change in CCT, CED, ACD and AL between two groups.

**Results:**

Mean age of patients was 56.23±5.22 years. Mean pre-operative CCT, CED, ACD and AL in PPV group was 533.06±13.28µm, 2231.67±164.57Cells/mm^2^, 3.37±0.18mm and 23.66±0.76mm respectively. Mean post-operative CCT, CED, ACD and AL in PPV group was 534.81±11.83µm, 2037.19±167.83 Cells/mm^2^, 3.24±0.13mm and 23.88±0.80mm respectively. Change in CED, ACD and AL from pre-operative value after PPV was statistically significant (p<0.001). Mean pre-operative CCT, CED, ACD and AL in SB group was 530.73±12.59 µm, 2161.79±161.96 Cells/mm^2^, 3.343±0.14mm and 23.67±0.82mm respectively. Mean post-operative CCT, CED, ACD and AL in SB group was 532.76±7.74 µm, 2158.27±156.58 Cells/mm^2^, 3.24±0.10mmand 25.71±0.86mm respectively. Change in ACD and AL from pre-operative value after SB was statistically significant (p<0.001). Mean change of CED, ACD and AL between two groups was also statistically significant (p<0.001).

**Conclusion:**

Rhegmatogenous retinal detachment repair causes ocular changes, with significantly more decrease in corneal endothelial cell density after pars plana vitrectomy, and more increase in anterior chamber depth and axial length after scleral buckling.

## INTRODUCTION

Retinal diseases have an immense impact on vision related quality of life. In resources limited countries like Pakistan, provision of primary health care facilities is a challenging task. In such conditions, the financial and socio-economic burden of provision of adequate retinal surgical facilities is a daunting assignment. Developing countries lack the training, equipment, and facilities for modern surgical management of retinal diseases.^1^ Rhegmatogenous Retinal Detachment (RRD) is an ophthalmic condition related to significant morbidity. It is detachment of neurosensory retina from retinal pigment epithelium, and is attributed to many risk factors like trauma, male gender and high myopia.^2^

The management of RRD was described initially in 1920 by Gonin, and has since then seen many microsurgical advancements and sophistications.^3^ Conventional management of RRD is Scleral Buckling (SB) which requires sealing of all retinal breaks by Cryotherapy and apposition of neurosensory retina with retinal epithelium using segmental or circumferential buckles.^4^ Other management option is Pars Plana Vitrectomy (PPV) introduced by Robert Machemer in 1970.^5^ This involves removal of vitreous, sealing of retinal breaks using endolaser and endotemponade of vitreous cavity using appropriate agent. The choices of these procedures rest on surgeon’s preference, phakic status, pupillary size, retinal breaks (number, size, type and location), and status of sub-retinal fluid and co-existent retinal or ophthalmic conditions. All these modern management options have led to marked improvement in results of RRD surgery from 0% to nearly 100%.^6^

These surgical options have their own advantages and disadvantages. Since the surgery has to affect long time visual prognosis, studies have been carried out to find the impact of these surgeries on multiple anatomical and physiological parameters of human eye. With variety of available non-invasive diagnostic modalities, it is easier to compare these two procedures in terms of their influence of different corneal and anatomical parameters. This study has been conducted to compare these two commonly performed procedures head to head, in order to help surgeons choose appropriate treatment option in variety of patients with co-existent ophthalmic conditions. The study will also explicate the changes surgeons should expect after retinal surgeries. The aim of the study was to compare the mean change in Central Corneal Thickness (CCT), Corneal Endothelial-Cell Density (CED), Anterior Chamber Depth (ACD) and Axial Length (AL) after repair of RRD by PPV and SB.

## METHODS

After approval by the hospital ethical review committee, informed written consent was taken from the patients prior to inclusion in the study. Phakic patients from either gender, aged between 40-70 years, with primary RRD were included. Patients with history of herpetic disease, keratoconus, glaucoma, pseudoexfoliation, cataract surgery, trauma, intravitreal injections or retinal lasers, vitreous hemorrhage, posterior uveitis, proliferative diabetic retinopathy and macular hole related RRD were excluded. Patients who failed to follow up for six months, and with post-operative complications like emulsified silicon oil in anterior chamber or pupillary block were also excluded. Demographic data of study population were acquired. All patients underwent complete ophthalmic examination including measurement of best corrected visual acuity, anterior segment examination to check for corneal pathologies or dystrophies and adequate pupillary dilatation. Pre-operative ACD and AL was measured using partial laser interferometry (IOL Master, Carl Zeiss Meditec, Dublin, CA, USA). CCT and CED were measured by taking mean of three readings with the help of Topcon SP 3000P Specular Microscope (Topcon Corporation, Tokyo, Japan). Pupils were then dilated with 0.1% topical Tropicamide eye drops. RRD was then examined using indirect ophthalmoscopy for type, location and number of retinal breaks, status of macula, extent of RRD and retinal degenerations.

All examination was performed by single retinal surgeon to exclude observer bias. Based on phakic status, morphology of retinal breaks, Proliferative Vitreo Retinopathy (PVR) and surgeon’s preference, the eyes underwent either SB or PPV. In Group-A, PPV was performed under local anesthesia. Standard 23 gauge, 3 ports PPV was performed. Breaks were sealed using photocoagulation, fluid air exchange was performed and endotemponade using C3F8 or Silicon oil. No SB was performed with PPV. In Group-B, SB was performed under general anesthesia. All breaks were identified using indirect ophthalmoscopy and indentation, and sealed using cryocoagulation. Encirclement or segmental band was then fixed 12–14 mm posterior to the limbus with 5–0 polybutylate coated polyester suture or silicon sleeve in case of encirclement band, avoiding the vortex veins. Trans-scleral drainage of sub-retinal fluid was done. No endotemponade was done with SB. All surgeries were performed by single retinal surgeon to exclude bias. The patients were regularly followed until six months, and the pre devised proforma was completed by single researcher endorsing subject’s demography and ocular examination findings on every visit. Final CCT, CED, ACD and AL were measured at six months. Those patients losing follow up were excluded.

### Statistical Analysis

Statistical package for social sciences (SPSS 17.0) for windows was used for statistical analysis. Descriptive statistics i.e. mean ± standard deviation for quantitative values (age, CCT, CED, ACD, AL) and frequencies along with percentages for qualitative variables (gender, laterality of eyes) were used to describe the data. Qualitative variables were compared using Chi Square test and quantitative variables were compared using independent ‘T’ test between two groups. Pre-operative and post-operative values within each group were compared using paired ‘t’ test. A p-value of <0.005 was considered statistically significant.

## RESULTS

A total of 81 eyes of 81 patients were initially included. Twelve patients lost follow up during study. Finally, 69 eyes of 69 patients were analyzed. 36 eyes underwent PPV (Group-A) and 33 eyes underwent SB (Group-B). In Group-A, 21 underwent endotemponade with 1000 centistokes silicon oil, while 15 underwent C3F8 endotemponade. In Group-B, 24 underwent SB using encirclement band, and 9 were operated with segmental band. Demographic data of the study population including age, gender, laterality, CCT, CED, ACD and AL is given in Table-I. Difference in age, gender, laterality and pre-operative CCT, CED, ACD and AL was not statistically significant between groups (p>0.005). Difference in post-operative CED, mean change in CED, mean change in ACD, AL and mean change in AL between groups was statistically significant (p=<0.005). Mean frequency increase in CCT was 0.32% and 0.38% in Group-A and Group-B respectively. In Group-A, 8.69% of endothelial cell loss was seen, as compared to 0.15% in Group-B. Mean frequency decrease in ACD was 3.56% and 5.38% in Group-A and Group-B respectively. Mean frequency increase in AL was 0.92% and 8.61% in Group-A and Group-B respectively. Within each group, mean pre-operative and post-operative CCT, CED, ACD and AL is given in Table-II. After surgery, difference of CED, ACD and AL from pre-operative value was statistically significant in Group-A (p=<0.001). In Group-B, difference of ACD and AL from pre-operative value was statistically significant (p=<0.001).

**Table-I:** Clinical Data of Study Population (n=69).

*Variable*	*Total n=69*	*Group-A (PPV Group) n=36*	*Group-B (SB Group) n=33*	p-Value* (Between groups)
Age (Years) mean ± SD	56.23±5.22	56.33 ± 5.09	56.12 ± 5.44	0.868
***Gender***				0.916
Male	36(52.17%)	19 (52.78%)	17(51.51%)	
Female	33(47.83%)	17 (47.22%)	16(48.49%)	
***Eye***				0.933
Right	38(55.07%)	20(55.55%)	18(54.54%)	
Left	31(44.93%)	16(44.45%)	15(45.46%)	
***CCT (µm) mean± SD***				
Pre-operative	531.94±12.92	533.06±13.28	530.73±12.59	0.459
Post-operative - 6 months	533.83±10.1	534.81±11.83	532.76±7.74	0.403
Mean change in CCT	1.88±7.53	1.75±6.48	2.03±8.64	0.879
***CED (Cells/mm^2^) mean ±SD***				
Pre-operative	2198.24±165.89	2231.67±164.57	2161.79±161.96	0.080
Post-operative – 6months	2095.10±172.47	2037.19±167.83	2158.27±156.58	0.003
Mean change in CED	-99.78±103.22	-194.47±35.42	-3.51±13.25	<0.001
***ACD (mm) mean ±SD***				
Pre-operative	3.39±0.16	3.37±0.18	3.34±0.14	0.130
Post-operative – 6months	3.24±0.12	3.24±0.13	3.24±0.10	0.94
Mean change in ACD	-0.15±0.09	-0.12±0.80	-0.18±0.85	0.002
***AL (mm) mean ±SD***				
Pre-operative	23.66±0.78	23.66±0.76	23.67±0.82	0.936
Post-operative – 6 months	24.76±1.24	23.88±0.80	25.71±0.86	<0.001
Mean change in AL	1.09±0.98	0.22±0.25	2.04±0.45	<0.001

* Independent ‘t’ Test

**Table-II:** Pre-op and Post-op Comparison between two groups.

*Variable*	*Group-A (PPV Group) n=36*	*Group-B (SB Group) n=33*
***CCT (µm) mean±SD***		
Pre-operative	533.06±13.28	530.73±12.59
Post-operative – 6 months	534.81±11.83	532.76±7.74
p-Value*	0.114	0.186
***CED (Cells/mm^2^) mean±SD***		
Pre-operative	2231.67±164.57	2161.79±161.96
Post-operative – 6 months	2037.19±167.83	2158.27±156.58
p-Value*	<0.001	0.137
***ACD (mm) mean±SD***		
Pre-operative	3.37±0.18	3.343±0.14
Post-operative – 6 months	3.24±0.13	3.24±0.10
p-Value*	<0.001	<0.001
***AL (mm) mean±SD***		
Pre-operative	23.66±0.76	23.67±0.82
Post-operative – 6 months	23.88±0.80	25.71±0.86
p-Value*	<0.001	<0.001

* Paired ‘t’ Test

## DISCUSSION

We observed minimal change in CCT in both PPV and SB group. This is contrary to the findings by Buettner H et al. who found that CCT increases significantly in all eyes after trans pars plana surgery.^7^ Another study found increase in CCT after PPV to be transient in nature only, with return to normal or pre-operative values one month after surgery.^8^ Similar findings were noted after SB too, where CCT was initially thickened after SB, but resumed its normal shape and thickness three months after SB.^9^ In a study, comparing change in CCT after PPV and SB with a follow up of 12 months, CCT changed from 560 ± 43µm to 548 ± 15 µm after 12 months in SB group, and from 554 ± 29 µm to 550 ± 19µm in PPV group. The comparison did not reveal a significant difference between two groups.^10^ In a recent study comparing corneal bio-chemical profiles after PPV and combined PPV and SB groups; both groups didn’t show any significant difference in corneal morphological parameters. They also concluded that SB doesn’t alter corneal morphology including CCT.^11^ Similar findings were found after PPV, where CCT did not change significantly during whole follow up period.^12^ We suggest that the findings of altered CCT are transient in nature only due to mild corneal edema, which eventually returns to normal or pre-operative values after both PPV and SB.

The effect of PPV or SB on CED is dependent on lens status. In our study, we included only phakic patients to compare these two methods to evaluate safety to corneal endothelium. We found 8.69% of endothelial cell loss after PPV, and 0.15% after SB. The difference in mean change in CED between two groups was statistically significant. Also, paired test revealed that as compared to pre-operative value, the CED significantly decreased in PPV group, but no significant change was seen in SB group. An estimated loss to endothelial cells averages around 8.5% after pars plana surgeries.^7^ Cinar E et al. found CED decrease to be 4.6±5.4% in phakic patients after silicon oil temponade. They also highlighted that endotemponade with either gas or silicon oil has almost same effect on CED decrease.^13^ An intact normal crystalline lens, or a stable iris-artificial lens diaphragm after cataract surgery is protective for corneal endothelium. More decrease in CED is noted in combined procedures involving anterior segment.^14^ Difference amongst researchers is found in estimated loss of endothelial cells after vitrectomy in phakic patients. One study found only 1.9% loss as compared to 1.8% in control group after PPV in phakic patients.^15^ Some studies have highlighted that endothelial loss after surgery is highest in immediate post-operative period, with loss being stabilized three to six months after surgery.^16^ One study found CED decrease to be 1.3 ± 1.4% in phakic eyes after PPV, but when combined with SB, mean cell loss was 8.5±1.8%. With additional fluid-gas exchange, a mean loss of 16.9 ±1.9% was noted.^17^ One study did not find any CED decrease after PPV in pseudophakic eyes.^18^ It is thus believed that an intact lens (either crystalline or artificial) is protective against endothelial damage. CED decease takes place more after PPV as compared to SB, and combined anterior segment surgeries can lead to further decrease in endothelial cell counts.

We observed significant decrease in ACD from pre-operative values in both groups. The decrease in SB group was significantly more as compared to PPV group. Increase or decrease in ACD after PPV is reported in literature. One study found increase in ACD after PPV and silicon oil temponade, and decrease in ACD after PPV with no temponade.^8^ Studies on SB have reported continued decrease in ACD three months after surgery.^9^ One study which compared ACD changes between PPV and SB reported more decrease in ACD after SB, as compared to PPV.^10^ One study found that ACD change may be transient in nature, with reduced levels until nine months, and eventual return to normal pre-operative values after one year.^19^ Wong CW et al found sustained decrease in ACD after one year follow period in eyes undergoing SB.^20^ We suggest that SB causes decrease in ACD which was present till six months, and longer follow up may reveal whether ACD remains decreased or not. As far as PPV is concerned, ACD changes are attributed to endotemponade, anterior shifting of iris-lens diaphragm and pupillary block that are dependent on type of temponading agent and other ophthalmic conditions.

Change in AL has long been reported after SB surgeries. Comparison of PPV and SB has revealed greater increase in AL after SB and minimal increase or stability of AL after PPV.^10^ Another study showed mean AL change of 0.95mm after SB, as compared to 0.1m after PPV.^21^ Studies have revealed AL change is dependent on type of SB and adjunctive treatment.^20^ A study on experimental animal model suggested more increase in AL observed after SB using band, as compared to sponge.^22^ Studies have even reported shortening of AL and hyperopic shift after local segmental SB, with myopic shift and increase in AL observed using encirclement SB.^23^ The increase in AL does not show progression, and stabilization of AL is noted 12 months after SB.^24^ We used encirclement SB in 24 patients and segmental SB in nine patients. A careful analysis revealed the similar findings, with more increase in AL observed after encirclement SB. However, no hyperopic shift or decrease in AL was observed with segmental band. The results are noted at six months, and further follow up will reveal stabilization of AL, or otherwise.

## CONCLUSION

Management of RRD by either PPV or SB results in changes in CED, ACD and AL. These factors must be considered when operating patients with co-existent corneal pathologies or axial myopia to avoid surgical or refractive surprise after surgery.

### Authors’ Contribution

**AM** conceived, designed and performed surgery.

**MAM** did data collection, statistical analysis, manuscript writing.

**ZUB** did statistical analysis and manuscript editing.

**MI** reviewed and finally approved manuscript.

## References

[1] Yorston D, Jalali S (2002). Retinal detachment in developing countries. Eye (Lond).

[2] Pandey AN, Kakde A (2014). A Retrospective Clinical Study of the Etiology and Post-operative Visual Outcome of Rhegmatogenous Retinal Detachment. J Clin Diagn Res.

[3] Mitry D, Awan MA, Borooah S, Rehman Siddiqui MA, Brogan K, Fleck BW (2012). Surgical Outcome and Risk stratification for Primary Retinal Detachment Repair. Br J Ophthalmol.

[4] Banaee T, Hosseini M, Ghooshkhanei H, Moosavi M, Kakhki K (2009). Anatomical and visual outcomes of three different sclera buckling techniques. J Ophth Vision Res.

[5] Jonas JB, Knorr HLJ, Rank RM, Budde WM (2001). Retinal redetachment after removal of intraocular silicone oil tamponade. Br J Ophthalmol.

[6] Hajari JN (2016). Optimizing the treatment of rhegmatogenous retinal detachment. Acta Ophthalmol.

[7] Buettner H, Bourne WM (1981). Effect of trans pars plana surgery on the corneal endothelium. Dev Ophthalmol.

[8] Calik B, Oztürk M, Serdarogullari H, Elcioglu M (2013). Evaluation of anterior segment parameters using pentacam in silicone oil-injected patients after pars plana vitrectomy. Indian J Ophthalmol.

[9] Cetin E, Ozbek Z, Saatci AO, Durak I (2006). The effect of scleral buckling surgery on corneal astigmatism, corneal thickness, and anterior chamber depth. J Refract Surg.

[10] Huang C, Zhang T, Liu J, Ji Q, Tan R (2016). Changes in axial length, central cornea thickness, and anterior chamber depth after rhegmatogenous retinal detachment repair. BMC Ophthalmol.

[11] Ruiz-De-Gopegui E, Ascaso FJ, Del Buey MA, Cristóbal JA (2011). Effects of encircling scleral buckling on the morphology and biomechanical properties of the cornea. Arch Soc Esp Oftalmol.

[12] Seymenoglu G, Uzun O, Başer E (2013). Surgically induced changes in corneal viscoelastic properties after 23-gauge pars plana vitrectomy using ocular response analyzer. Curr Eye Res.

[13] Cinar E, Zengin MO, Kucukerdonmez C (2015). Evaluation of corneal endothelial cell damage after vitreoretinal surgery:comparison of different endotamponades. Eye (Lond).

[14] Goezinne F, Nuijts RM, Liem AT, Lundqvist IJ, Berendschot TJ, Cals DW (2014). Corneal endothelial cell density after vitrectomy with silicone oil for complex retinal detachments. Retina.

[15] Matsuda M, Tano Y, Inaba M, Manabe R (1986). Corneal endothelial cell damage associated with intraocular gas tamponade during pars plana vitrectomy. Jpn J Ophthalmol.

[16] Takkar B, Jain A, Azad S, Mahajan D, Gangwe BA, Azad R (2014). Lens status as the single most important factor in endothelium protection after vitreous surgery:a prospective study. Cornea.

[17] Friberg TR, Doran DL, Lazenby FL (1984). The effect of vitreous and retinal surgery on corneal endothelial cell density. Ophthalmology.

[18] Mittl RN, Koester CJ, Kates MR, Wilkes E (1989). Endothelial cell counts following pars plana vitrectomy in pseudophakic and aphakic eyes. Ophthalmic Surg.

[19] Goezinne F, La Heij EC, Berendschot TT, Tahzib NG, Cals DW, Liem AT (2010). Anterior chamber depth is significantly decreased after scleral buckling surgery. Ophthalmology.

[20] Wong CW, Ang M, Tsai A, Phua V, Lee SY (2016). A Prospective Study of Biometric Stability After Scleral Buckling Surgery. Am J Ophthalmol.

[21] Brazitikos PD, Androudi S, Christen WG, Stangos NT (2005). Primary pars plana vitrectomy versus scleral buckle surgery for the treatment of pseudophakic retinal detachment:a randomized clinical trial. Retina.

[22] Moshfeghi AA, Pendergast SD, Hartzer MK, Ferrone PJ (2004). The effects of scleral buckling on young rabbit eyes. Arch Ophthalmol.

[23] Okada Y, Nakamura S, Kubo E, Oishi N, Takahashi Y, Akagi Y (2000). Analysis of changes in corneal shape and refraction following scleral buckling surgery. Jpn J Ophthalmol.

[24] Malukiewicz-Wisniewska G, Stafiej J (1999). Changes in axial length after retinal detachment surgery. Eur J Ophthalmol.

